# Autophagic flux disruption contributes to *Ganoderma lucidum* polysaccharide-induced apoptosis in human colorectal cancer cells via MAPK/ERK activation

**DOI:** 10.1038/s41419-019-1653-7

**Published:** 2019-06-11

**Authors:** Haitao Pan, Yujie Wang, Kun Na, Ying Wang, Lu Wang, Zhenhao Li, Chengjie Guo, Dandan Guo, Xingya Wang

**Affiliations:** 1 Department of Pharmaceutical Science, Zhejiang Chinese Medical University, 548 Binwen Road, 310053 Hangzhou, Zhejiang China; 2 Zhejiang Shouxiangu Institute of Rare Medicine Plant, 12, Huanglong 3rd Road, 321200 Wuyi, Zhejiang China

**Keywords:** Cancer prevention, Cancer therapy, Colorectal cancer, Macroautophagy, Macroautophagy

## Abstract

Targeting autophagy may serve as a promising strategy for cancer therapy. *Ganoderma lucidum* polysaccharide (GLP) has been shown to exert promising anti-cancer effects. However, the underlying mechanisms remain elusive. Whether GLP regulates autophagy in cancer has never been reported. In this study, GLP induced the initiation of autophagy in colorectal cancer (CRC) HT-29 and HCT116 cells, as evidenced by enhanced level of LC3-II protein, GFP-LC3 puncta, and increased formation of double membrane vacuoles. However, GLP treatment caused marked increase of p62 expression. Addition of late stage autophagy inhibitor, chloroquine (CQ), further enhanced LC3-II and p62 level, as well as increased autophagosome accumulation, suggesting a blockage of autophagic flux by GLP in CRC cells. We then found GLP blocked autophagosome and lysosome fusion as determined by mRFP-GFP-LC3 colocalization analysis. Mechanistic study revealed that GLP-induced disruption of autophagosome-lysosome fusion is due to reduced lysosome acidification and lysosomal cathepsin activities. Cell viability and flow cytometry assays revealed that GLP-induced autophagosome accumulation is responsible for GLP-induced apoptosis in CRC cells. In line with this, inhibition of autophagy initiation by 3-methyladenine (3-MA), an early stage autophagy inhibitor, attenuated GLP-induced apoptosis. In contrast, suppression of autophagy at late stage by CQ enhanced the anti-cancer effect of GLP. Furthermore, we demonstrated that GLP-induced autophagosome accumulation and apoptosis is mediated via MAPK/ERK activation. Finally, GLP inhibited tumor growth and also inhibited autophagic flux in vivo. These results unveil new molecular mechanism underlying anti-cancer effects of GLP, suggesting that GLP is a potent autophagy inhibitor and might be useful in anticancer therapy.

## Introduction

Colorectal cancer (CRC) is the third most common malignancy and the third leading cause of cancer-related death in the USA^1^. Despite the benefits of early screening, surgery, and other therapeutic interventions, the current five-year survival rate for late stage CRC patients is still low^2^. Therefore, the search for effective and safe agents for CRC prevention and treatment is still urgently needed.

Natural products with highly diverse bioactivities and functions play a vital role in drug discovery for treating diseases. *Ganoderma lucidum* (*G. lucidum*) is a medicinal mushroom that has been used in East Asian countries for over 2000 years^3–5^. A substantial number of preclinical and clinical studies have reported that *G. lucidum* has numerous pharmacological effects, including antioxidant, hypoglycemic, immune-regulatory, anti-diabetic, and anti-cancerous^5–10^. Many studies have demonstrated that GLP is one of the main bioactive components responsible for anti-cancer effects of *G. lucidum*^3,5^. Recently, we showed that GLP extracted from the sporoderm-broken spores of *G. lucidum* significantly inhibited cell proliferation and induced apoptosis in colorectal and prostate cancer cells^11,12^. However, the molecular mechanisms underlying the anti-cancer effects of GLP remain unclear.

Autophagy is an evolutionarily conserved catabolic process that degrades cytoplasmic materials and provides substrates for energy metabolism during nutrient deprivation and metabolic stress^13^. Autophagy has been closely related to many human diseases, including obesity, aging, neurodegenerative disorders, and cancer^13^. The role of autophagy in cancer is complex and differs among various types of cancer^14,15^. Autophagy inhibits tumor initiation and progression in some cancers, but promotes tumor survival and progression in others^14,15^. Given these dual effects, therapeutic modulation of autophagy may serve as promising but challenging means for cancer treatment. Autophagy is considered a second type of programmed cell death (PCD)^16^. Intriguingly, it has been proposed that the interplay between autophagy and apoptosis, the type I PCD, may contribute to the anti-cancer effects of many anti-cancer agents^17,18^. However, what molecules or signaling pathways mediate the crosstalk between autophagy and apoptosis, whether these two PCDs regulate each other, and how anti-cancer agents affect these processes remain elusive.

In this study, we sought to examine the effect of GLP on autophagy and to evaluate whether such effect is relevant to the apoptotic effect induced by GLP in CRC, which has never been reported before. We found that GLP served as an autophagy initiation inducer and also a novel autophagic flux inhibitor by interfering with autophagosome-lysosome fusion. In addition, GLP-induced autophagosome accumulation is required for GLP-induced apoptosis in CRC cells. Furthermore, we demonstrated that GLP-induced autophagosome accumulation and apoptosis is mediated by MAPK/ERK activation.

## Results

### GLP inhibits cell viability and induces autophagy initiation in CRC cells

**Fig. 1:**
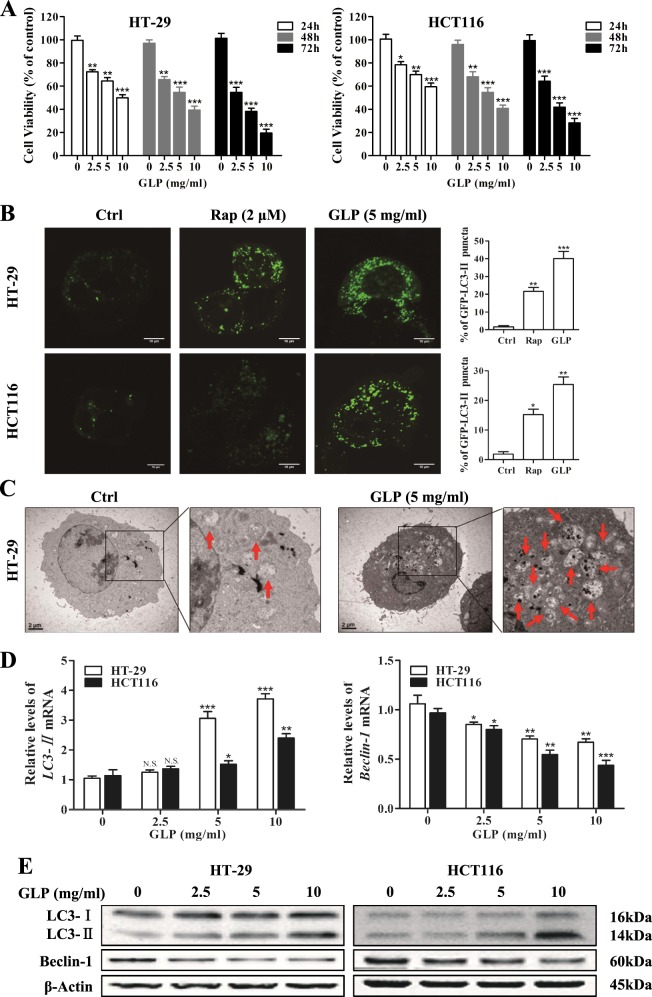
GLP inhibits cell viability and induces autophagy initiation in CRC cells. **a** HT-29 and HCT116 cells were treated with indicated concentrations of GLP for 24, 48, and 72 h. Cell viability was measured by the MTT assay. **b** HT-29 and HCT116 cells were transfected with GFP-LC3 adenovirus for 24 h, and treated with GLP (5 mg/ml) and Rap (2 μM) for another 24 h. GFP-LC3 puncta was visualized by confocal microscope. The number of GFP-LC3 puncta per cell was quantified and presented as mean ± SE from 100 randomly selected cells (*n* = 3). Scale bar: 10 µm. **c** Transmission electron microscopy (TEM) was utilized to observe the formation of autophagosome upon GLP treatment (24 h). Arrows indicate representative double membrane vacuoles. Scale bar: 2 µm. **d** qRT-PCR determination of the mRNA expression of LC3 and Beclin-1 in HT-29 and HCT116 cells upon treatment with GLP at indicated concentrations for 24 h. **e** Western blotting analysis of LC3 and Beclin-1 expression in HT-29 and HCT116 cells upon GLP treatment (24 h). Data are presented as the mean ± SE from three independent experiments. *P < 0.05; **P < 0.01; ***P < 0.001 compared with untreated cells. N.S. No significance

We first examined the effect of GLP on cell viability in HT-29 and HCT116 cells by MTT assay. As shown in Fig. 1a, GLP significantly reduced cell viability in both cells. In order to examine the effect of GLP on autophagy, we evaluated the distribution pattern of GFP-LC3 in CRC cells transiently expressing GFP-LC3, reminiscent of autophagosome formation^19^. During autophagy, the cytoplasmic form LC3-I is modified to LC3-II, thus, the amount of LC3-II increases with the formation of autophagosomes^19^. As shown in Fig. 1b, GLP-treated cells exhibited a dramatic increase in the punctuate distribution of GFP-LC3 in CRC cells, whereas autophagy inducer rapamycin (Rap) treated cells displayed less distribution of puncta. Quantitative analysis further confirmed this observation (Fig. 1b). We next confirmed the induction of autophagy initiation by GLP using transmission electron microscopy (TEM) in HT-29 cells. After treating cells with GLP for 24 h, numerous double-membrane autophagic vacuoles were observed in HT-29 cells, but much less in untreated cells (Fig. 1c).

Furthermore, GLP induced LC3-II expression at both mRNA and protein levels in a dose-dependent manner in CRC cells (Fig. 1d, e). However, the expression of Beclin-1, a key regulator of autophagy^20^, was down-regulated by GLP at both the protein and mRNA levels (Fig. 1d, e), suggesting that GLP-induced autophagy is Beclin-1-independnt in CRC cells. Supplementary Fig. 1 shows densitometric analysis of western blots.

### GLP inhibits autophagic flux in CRC cells

**Fig. 2:**
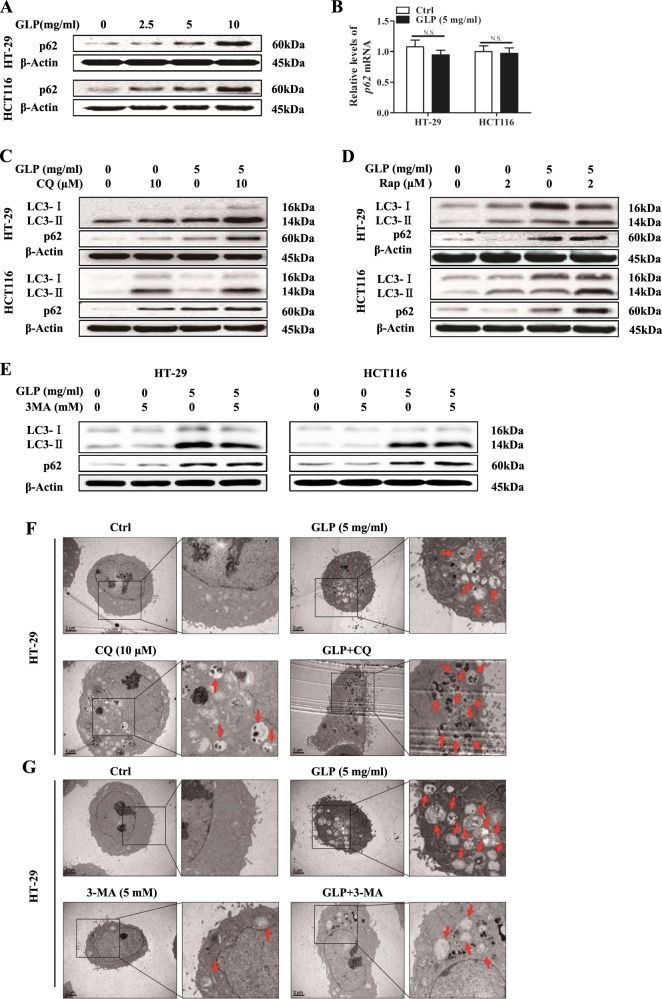
GLP inhibits autophagic degradation and increases autophagosome formation in CRC cells. **a** HT-29 and HCT116 cells were treated with GLP at indicated concentrations for 24 h, and the expression of p62 was determined by western blotting analysis. **b** qRT-PCR analysis of the mRNA expression level of p62 following treatment of HT-29 and HCT116 cells with GLP (5 mg/ml) for 24 h. **c**–**e** HT-29 and HCT116 cells were treated with GLP (5 mg/ml), with or without CQ (10 μM) (**c**), or with or without Rap (2 μM) (**d**), or with or without 3-MA (5 mM) (**e**) for 24 h. The protein expressions of LC3 and p62 were determined by western blotting. **f**, **g** HT-29 cells were treated with GLP (5 mg/ml), with or without CQ (10 μM) (**f**), or with or without 3-MA (5 mg/ml) (**g**) for 24 h. TEM was utilized to observe the formation of autophagosome upon treatments. Arrows indicate representative double membrane vacuoles. Scale bar: 2 µm. Data are presented as Mean ± SE from three independent experiments. N.S. No significance

The assessment of autophagy activity is not only dependent on the measurement of autophagy initiation, but also the degradation of autophagy substrates^21^. The accumulation of sequestosome-1 or p62, an autophagic cargo protein, inversely correlates with autophagic activity^22^. Our results showed that p62 expression was significantly increased upon GLP treatment in CRC cells at translational but not transcriptional levels (Fig. 2a, b), suggesting impaired autophagy flux occurred.

The effect of GLP on autophagy flux was evaluated by treating cells with late stage autophagy flux inhibitor CQ. As seen in Fig. 2c, both CQ (10 µM) and GLP (5 mg/ml) alone, significantly increased the level of LC3-II and p62 in CRC cells. Moreover, GLP-induced accumulation of LC3-II and p62 was further enhanced by CQ treatment (Fig. 2c), suggesting a synergistic effect on autophagy flux inhibition. In contrast, the autophagy initiation inducer, Rap, upregulated the level of LC3-II but decreased p62 expression in CRC cells, indicating normal autophagy proceeded (Fig. 2d). However, Rap further enhanced GLP-induced LC3-II and p62 expression in both cells, suggesting a synergistic effect with GLP on inducing autophagic autophagosome accumulation (Fig. 2d). We also examined the effect of autophagy early stage inhibitor 3-MA on GLP-induced LC3-II and p62 accumulation in CRC cells. As expected, 3-MA alone slightly inhibited LC3-II, increased p62 expression and further reduced GLP-induced LC3-II expression, but increased p62 expression significantly in HT-29 cells (Fig. 2e). Despite 3-MA significantly reduced GLP-induced LC3-II upregulation and further increased GLP-induced p62 expression in HCT116 cells, it also slightly increased LC3-II expression and reduced p62 expression when treated alone (Fig. 2e). These data suggest that 3-MA may play a complex role in regulating autophagy in these two CRC cells. Supplementary Fig. 2 shows densitometric analysis of western blots.

TEM analysis further confirmed a dramatic increase in the number of autophagic vacuoles upon GLP or CQ treatment in HT-29 cells, and a synergistic effect upon co-treatment with both agents (Fig. 2f). In contrast, 3-MA which inhibited the initiation of autophagy, formed very few autophagic vacuoles than GLP-treated cells (Fig. 2g). Consistent with results from western blotting, 3-MA dramatically reduced GLP-induced vacuole formation in HT-29 cells (Fig. 2g). Taken together, these results suggest that GLP-induced autophagosome accumulation is due to both the initiation of autophagy and inhibition of autophagic flux.

### GLP blocks autophagosome-lysosome fusion in CRC cells

**Fig. 3:**
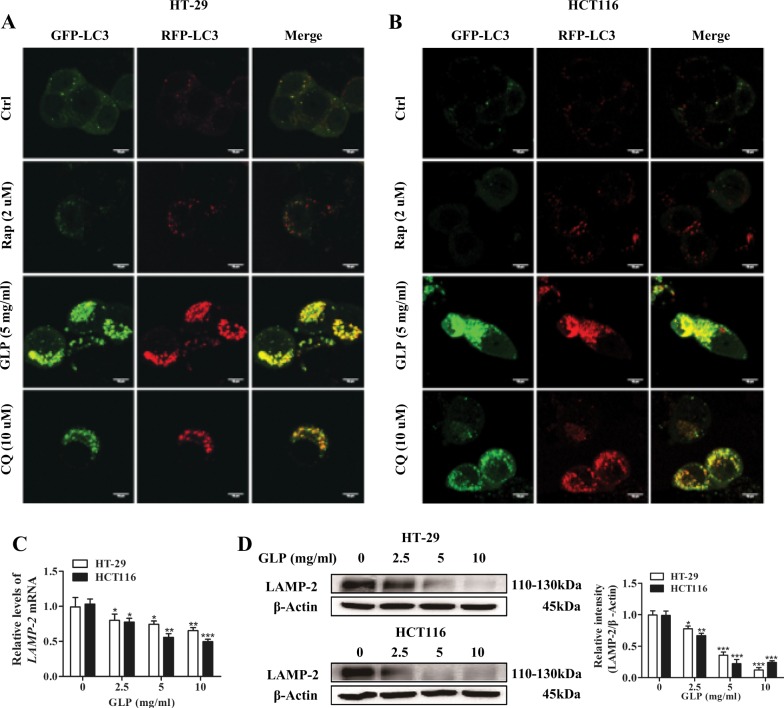
GLP inhibits autophagosome-lysosome fusion in CRC cells. **a**, **b** HT-29 (**a**) and HCT116 (**b**) cells were transfected with mRFP-GFP-LC3 adenovirus and treated with Rap (2 μM), GLP (5 mg/ml), or CQ (10 μM) for 24 h, respectively. Cells were observed to distinguish between autophagosome (yellow puncta) and autolysosome (red puncta) after colocalization analysis using a confocal microscope. Scale bar: 10 µm. **c** Quantification of the mRNA expression level of LAMP-2 in HT-29 and HCT116 cells upon treatment with GLP at indicated concentrations for 24 h. **d** Western blotting analysis of LAMP-2 protein expression in CRC cells upon GLP treatment for 24 h. The relative intensity of LAMP-2 was calculated after normalization against β-Actin. Data are presented as the mean ± SE from three independent experiments. *P < 0.05; **P < 0.01; ***P < 0.001 compared with untreated cells

The fusion of autophagosome and lysosome is important for autophagic flux, and inhibition of this process impairs autophagic degradation^22^. We transfected CRC cells with mRFP-GFP-LC3 adenovirus that serves as a dual-fluorescence pH sensor for autophagic vacuoles and assessed autolysosome formation. As shown in Fig. 3a, treatment with Rap enhanced both green and red dot formation and colocalization gave rise to more red-only puncta, suggesting autolysosome maturation proceeded normally upon Rap treatment in HT-29 and HCT116 cells. Conversely, both the lysosomal acidification inhibitor CQ and GLP increased red and green puncta in CRC cells dramatically, while the yellow punctate fluorescence increased markedly after colocalization, indicating CQ and GLP blocked autophagosome-lysosome fusion in these cells (Fig. 3a, b). Notably, GLP yielded more yellow punctate fluorescence than CQ.

To confirm the above observation, we examined the expression of autophagosome-lysosome fusion marker, lysosome-associated membrane protein 2 (LAMP-2) in CRC cells. Indeed, we found that GLP significantly reduced the expression of LAMP-2 at both mRNA and protein levels in CRC cells (Fig. 3c, d). These results suggest that GLP-induced autophagic flux inhibition in CRC cells is due to impaired autophagosome-lysosome fusion.

### GLP inhibits lysosomal acidity and cathepsin activity

**Fig. 4:**
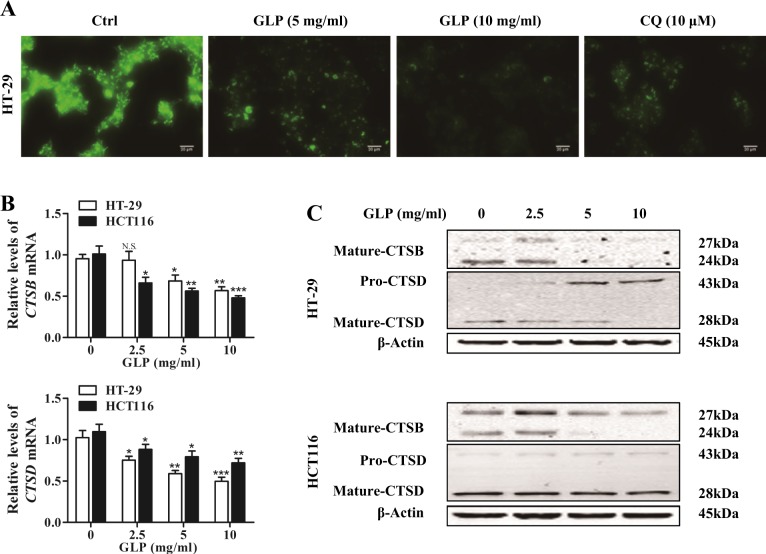
GLP inhibits lysosome acidity and impairs lysosomal function in CRC cells. **a** Inhibition of lysosome acidification upon treatment with GLP (5, 10 mg/ml) and CQ (10 μM) for 24 h in HT-29 cells as determined by LysoSensor Green DND-189 (1 mM) staining. Representative fluorescence microscopy images are shown. Scale bar: 20 µm. **b** Quantification of the mRNA expression levels of lysosomal proteases, CSTB and CTSD, in HT-29 and HCT116 cells upon GLP treatment for 24 h at indicated concentrations. **c** Western blotting analysis of pro-form and mature-form of CTSB and CTSD proteins in CRC cells upon GLP treatment for 24 h at indicated concentrations. Data are presented as the mean ± SE from three independent experiments. *P < 0.05; **P < 0.01; ***P < 0.001 compared with untreated cells. N.S. No significance

Lysosomal enzymes play an important role in autophagic degradation, and the function of lysosomal enzymes are dependent on a narrow range of acidic pH^23^. We next assessed the effect of GLP on lysosome acidification by staining HT-29 cells with LysoSensor Green DND-189, an acidotropic fluorescent probe that can be trapped in acidic organelles, such as lysosomes. As shown in Fig. 4a, GLP significantly reduced the green fluorescence intensity in HT-29 cells, which is similar to CQ, the positive control. This result indicates that GLP inhibits lysosomal acidity in CRC cells.

Autophagy is a process by which the cytoplasmic components are degraded by proteases in lysosomes^24^. We then investigated whether GLP treatment affects the expression of lysosomal cathepsin B and D (CSTB and CSTD). Both cathepsins are produced from precursor forms (pro-cathepsins) into the mature-cathepsins^25,26^. As shown in Fig. 4b, GLP significantly reduced the expression of CTSB and CTSD at mRNA level. In addition, the expression of the mature-CTSB and mature-CTSD at protein levels was also reduced in CRC cells (Fig. 4c). Taken together, the above results suggest that GLP reduces the acidity of lysosome and inhibits lysosomal cathepsin activities in CRC cells. Supplementary Fig. 3 shows densitometric analysis of western blots.

### GLP induces autophagosome formation by activating MAPK/ERK signaling

**Fig. 5:**
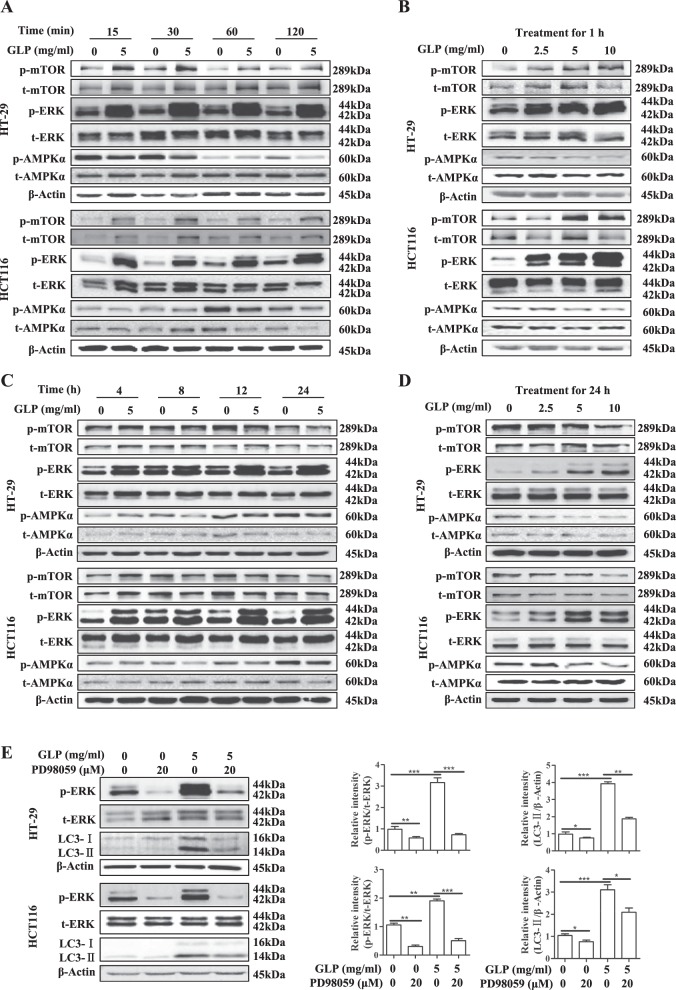
Effect of GLP on mTOR, AMPK, and MAPK/ERK signaling pathways in CRC cells. **a** HT-29 and HCT116 cells were treated with or without GLP (5 mg/ml) for 15, 30, 60, and 120 min. **b** HT-29 and HCT116 cells were treated with 5 mg/ml of GLP for 1 h at indicated concentrations. **c** HT-29 and HCT116 cells were treated with GLP (5 mg/ml) for 4, 8, 12, and 24 h. **d** HT-29 and HCT116 cells were treated with 5 mg/ml of GLP for 24 h at indicated concentrations. **e** HT-29 and HCT116 cells were pre-treated with PD98059 (20 µM) for 2 h, then co-treated with GLP (5 mg/ml) for additional 24 h. (For **a**–**d**, the expressions of both phosphorylated and total mTOR, AMPKα, and MAPK/ERK were determined by western blotting. The relative intensities of LC3-II and p-MAPK/ERK were calculated after normalization against β-Actin and t-MAPK/ERK, respectively. Data are presented as the Mean ± SE from three independent experiments. *P < 0.05; **P < 0.01; ***P < 0.001 compared with untreated cells

Autophagy is also regulated by many signaling pathways, including mTOR, AMPK, and MAPK^27^. We next examined the role of these signaling pathways in GLP-induced autophagic deregulation. As shown in Fig. 5a, GLP (5 mg/ml) transiently induced the phosphorylation of mTOR and MAPK/ERK, but reduced AMPKα phosphorylation within 2 h in CRC cells. In addition, GLP dose dependently induced mTOR and MAPK/ERK phosphorylation, but reduced AMPKα phosphorylation in CRC cells when treated for 1 h (Fig. 5b). We then extended time of treatment, and found GLP-induced MAPK/ERK phosphorylation remained high at 24 h (Fig. 5c). The result of p-AMPKα is confusing which did not show a clear trend upon prolonged treatment. Interestingly, the phosphorylation of mTOR was not further increased by GLP at 4 and 8 h in both cells, but started to decrease at 12 h in CRC cells and maintained to be inhibited at 24 h (Fig. 5c). Furthermore, at 24 h, GLP inhibited the phosphorylation of mTOR and AMPKα, but increased the phosphorylation of MAPK/ERK in a dose-dependent manner in both cells (Fig. 5d).

Previous studies have demonstrated that MAPK/ERK is an important regulator of autophagy^28^. In order to examine whether activated MAPK/ERK mediates GLP-induced autophagy in CRC cells, we pre-treated both cells with 20 µM of PD98059, a MAPK/ERK inhibitor, for 2 h and then treated with GLP for additional 24 h. Western blotting analysis revealed that the expression of GLP-induced LC3-II was significantly downregulated by PD98059 in CRC cells (Fig. 5e), suggesting that MAPK/ERK activation is responsible for GLP-induced autophagy initiation. Supplementary Fig. 4 shows densitometric analysis of western blots.

### Accumulation of autophagosome confers cytotoxicity in CRC cells

**Fig. 6:**
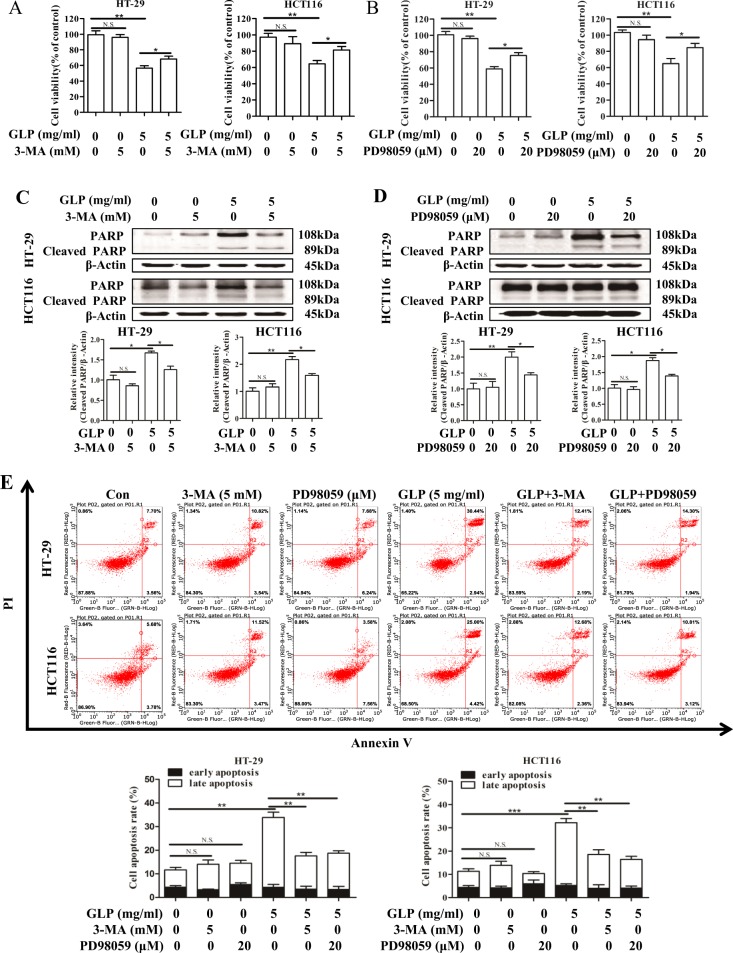
Inhibition of autophagosome accumulation and MAPK/ERK activation rescues GLP-induced cell death and apoptosis in CRC cells. **a**, **b** HT-29 and HCT116 cells were treated with GLP (5 mg/ml) with or without 3-MA (5 mM) or with or without PD98059 (20 μM) for 24 h. The cell viability was then determined by MTT assay. **c**, **d** HT-29 and HCT116 cells were treated with GLP (5 mg/ml) with or without 3-MA (5 mM) (**c**), or with or without PD98059 (**d**) for 24 h. The expression of PARP was determined by western blotting. The relative intensities of cleaved-PARP in each cell line were calculated after normalization against β-Actin. **e** HT-29 and HCT116 cells were treated with GLP (5 mg/ml) with or without 3-MA (5 mM), or with or without PD98059 (20 μM) for 24 h. Apoptosis was analyzed by flow cytometry. Percentage of total apoptotic cells with both early and late apoptosis was calculated and presented. Data are presented as the mean ± SE from three independent experiments. *P < 0.05; **P < 0.01; ***P < 0.001. N.S. No significance

We then examined whether autophagosome accumulation and MAPK/ERK activation play roles in GLP-induced cytotoxicity and apoptosis in CRC cells. As shown in Fig. 6a, b, addition of 3-MA or PD98059 significantly increased GLP-reduced cell viability in CRC cells. Furthermore, results from western blotting and flow cytometry showed that GLP alone significantly induced poly (ADP-ribose) (PARP) cleavage and apoptosis in both cells (Fig. 6c–e). However, both 3-MA and PD98059 significantly rescued GLP-induced PARP cleavage and apoptosis in CRC cells (Fig. 6c–e).

**Fig. 7:**
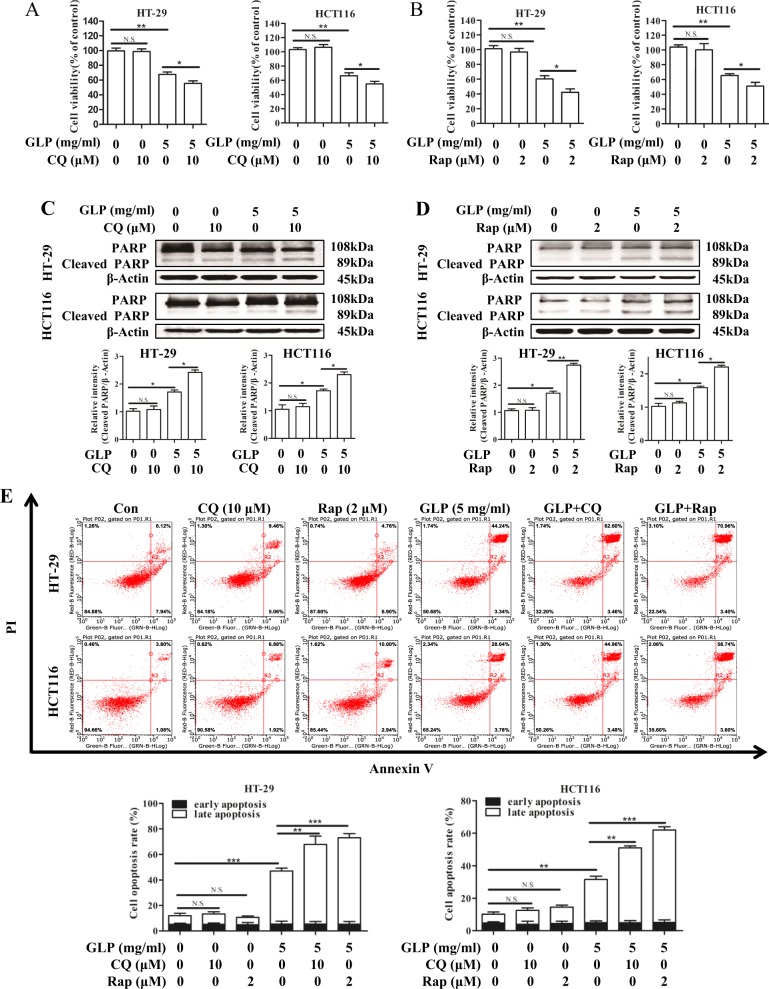
Increasing autophagosome accumulation confers GLP-induced cell death and apoptosis in CRC cells. **a**, **b** HT-29 and HCT116 cells were treated with GLP (5 mg/ml) with or without CQ (10 μM), or Rap (2 μM) for 24 h. The cell viability was then determined by MTT assay. **c**, **d** HT-29 and HCT116 cells were treated with GLP (5 mg/ml) with or without CQ (10 μM) (**c**), or with or without Rap (2 μM) (**d**) for 24 h. The expression of PARP was determined by western blotting. The relative intensities of cleaved-PARP in each cell line were calculated after normalization against β-Actin. **e** HT-29 and HCT116 cells were treated with GLP (5 mg/ml), with or without CQ (10 μM) or with or without Rap (2 μM) for 24 h. Apoptosis was analyzed by flow cytometry. Percentage of total apoptotic cells with both early and late apoptosis was calculated and presented. Data are presented as the mean ± SE from three independent experiments. *P < 0.05; **P < 0.01; ***P < 0.001. N.S. No significance

Furthermore, we analyzed the effect of increasing the accumulation of autophagosomes on cell viability and apoptosis upon GLP treatment in CRC cells. As shown in Fig. 7a, b, both the late stage autophagy inhibitor CQ and early stage autophagy inducer Rap, which increase autophagosome accumulation, significantly enhanced GLP-induced cell death in both cells. In addition, GLP-induced PARP cleavage and apoptosis were further increased by CQ or Rap (Fig. 7c–e). Taken together, these results demonstrate that GLP-induced autophagic flux disruption, and thus autophagosome accumulation contributes to GLP-induced cytotoxicity and apoptosis in CRC cells, which maybe mediated via MAPK/ERK activation in CRC cells.

### GLP inhibits tumor growth and inhibits autophagy in vivo

**Fig. 8:**
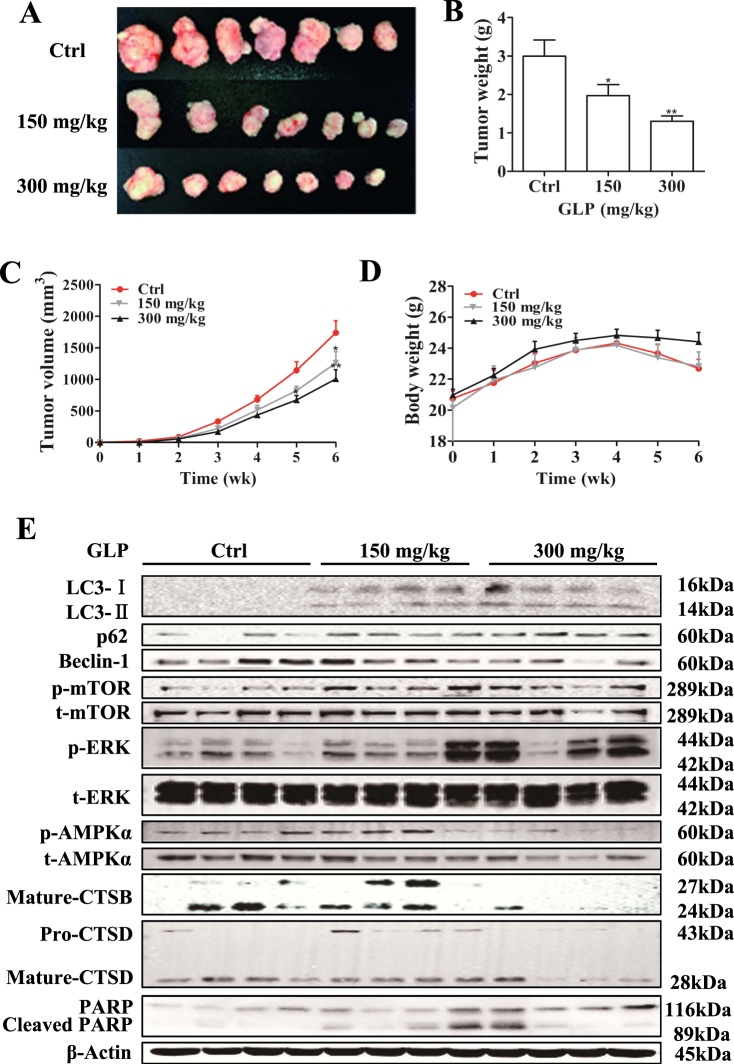
GLP inhibits tumor growth and inhibits autophagy in HT-29 xenograft nude mice. **a** Image of the tumor samples from each group at necropsy: control; 150 mg/kg and 300 mg/kg. **b** Average final tumor weight of each group. **c** Growth curve of tumor volume in nude mice. **d** Effect of GLP treatment on body weight of nude mice. **e** Expression of LC3, p62, Beclin-1, p-mTOR, p-AMPKα, p-MAPK/ERK, Mature-CTSB, and Mature-CTSD in xenograft tumor at protein levels was determined by western blotting. β-Actin used as internal control. Data are presented as the mean ± SE from 7 xenograft tumor samples of each group. *P < 0.05; **P < 0.01; ***P < 0.001 compared with control group

To determine the antitumor activity of GLP in vivo, male BALB/C nude mice were injected with HT-29 cells subcutaneously and then treated with GLP (150 mg/kg and 300 mg/kg). As shown in Fig. 8a–d, GLP treatment significantly decreased tumor volume and weight compared with control group, while the body weight of mice was not affected by GLP treatments. Next, we examined the regulation of autophagy by GLP in tumor samples. As shown in Fig. 8e, consistent with in vitro experiments, GLP increased the levels of LC3-II, p62, p-MAPK/ERK, and decreased the levels of Beclin-1 and p-AMPKα in xenograft tumors. Moreover, the levels of mature-CTSB and mature-CTSD were decreased in tumor sample from high-dose treatment (Fig. 8e). However, the expression of p-mTOR was increased in GLP-treated tumor samples (Fig. 8e). We also examined the apoptosis index by terminal deoxynucleotidyl transferase dUTP nick end labeling (TUNEL) staining. Unfortunately, GLP did not significantly increase apoptosis in xenograft tumors (Supplementary Fig. 5), though western blotting analysis showed that PARP cleavage might be higher in GLP-treated tumor samples than controls (Fig. 8e). These results indicate that GLP inhibits tumor growth and autophagy flux in vivo, but the role of GLP-induced autophagy inhibition in the tumor growth in xenografts is unknown in vivo.

## Discussion

The role of autophagy in cancer is complex and context dependent^29^. Overwhelming preclinical and clinical evidence suggests that suppression of autophagy effectively inhibit cancer development and often sensitizing cancer cells to chemotherapeutic agents^30–32^. CQ and its analog, hydroxychloroquine (HCQ), are the only autophagy inhibitor being used for treating cancer in clinical trials^33^. However, Phase I/II clinical trials with CQ and HCQ showed mixed results with varying levels of efficacy, highlighting the needs to develop alternative autophagy inhibitors^33–35^. Numerous studies have indicated that GLP inhibits tumorigenesis through inducing apoptosis, inhibiting invasion, and regulating immune response^3,5,8^. However, there has been no report regarding the effect of GLP on autophagy in cancer. As summarized in Supplementary Fig. 6, our study demonstrates that GLP induced autophagy initiation and suppressed autophagic degradation through inhibiting lysosomal acidification and downregulating lysosomal enzymes. The accumulation of autophagosomes that resulted from autophagic flux disruption contributes to GLP-induced apoptosis through MAPK/ERK activation in CRC cells.

Although defined as type II PCD, controversy exists over whether autophagy alone can drive cell death and the molecular mechanisms underlying autophagic cell death are poorly understood^16,36^. Autophagy and apoptosis could cross-regulate each other and often occur in a sequence in which autophagy precedes apoptosis^17^. In line with our results, bodies of evidence suggest that disruption of the late stage of autophagy leads to excessive accumulation of autophagosomes, which has the potential to turn autophagy into a destruction process and induce apoptosis^37–41^. This notion is further supported by results obtained after adding Rap to CRC cells in our study. The autophagy inducer Rap induced normal process of autophagy and showed no effect on apoptosis in CRC cells. However, apoptosis was enhanced when cells were co-treated with GLP and Rap, suggesting that autophagosome accumulation plays a key role in GLP-induced cell death. In addition, blocking of autophagosome formation by autophagy in early stage inhibitor 3-MA dramatically rescued GLP-induced apoptosis. In contrast, the late stage autophagy inhibitor CQ which increases autophagosome formation, enhanced GLP-induced cytotoxicity and apoptosis. These results suggest that GLP-induced autophagosome accumulation is responsible for its pro-apoptotic effect in CRC cells. Similarly, a substantial number of studies have shown that other chemotherapy drugs induced the accumulation of autophagosomes due to autophagic flux disruption also confers cytotoxicity^21,42–46^.

Using both genetic and chemical approaches, Button et al. found defects in autophagosome-lysosome fusion or lysosome alone do not induce sufficient cellular toxicity. However, it was the increased autophagosome synthesis and accumulation that directly induce cell death in three different cancer cell models^45^. Together with our results, autophagosome accumulation through inhibiting autophagy may be a valuable strategy for cancer therapy by anti-cancer agent, such as GLP. However, in neurodegenerative or other diseases, lowering autophagosome accumulation may have a therapeutic value instead^47^.

Autophagy and apoptosis are under the control of multiple molecules and signaling pathways^16^. The classical regulation of autophagy is governed by the mTOR pathway, upon inhibition by AMPK^27^. However, apart from classical role of mTOR, numerous studies have reported that autophagy induction could also be mTOR-independent process^48–51^. Furthermore, some studies found that inhibition of mTOR would rather impair the autophagic flux, thus leads to enhanced autophagosome accumulation rather than autophagy induction^52,53^. These observations may support our findings that a later inhibition (after 8 h) of mTOR might be important in GLP-induced autophagy flux disruption. In our study, we also found that the regulation of autophagy by GLP may be AMPK-independent, as supported by studies from others^54,55^. Interestingly, Vucicevic et al. reported that Compound C, a specific AMPK inhibitor, actually inhibited autophagy through an AMPK-independent inhibition of mTOR in cancer cells^56^.

Beclin-1 is essential for the formation and extension of pre-autophagosomal structure^20^. However, numerous studies suggest that there are Beclin-1-independent mechanisms of autophagy and autophagy-independent roles for Beclin-1 in cancer^57–63^. Our study showed that the accumulation of autophagosomes induced by GLP is not accompanied by the upregulation of Beclin-1, but rather Beclin-1 is inhibited by GLP in CRC cells. Although suggested as a tumor suppressor gene, role of Belin-1 in cancer is very complicated and controversial^64^. Recent studies reported that deletion of Beclin-1 expression by RNAi promoted apoptosis in CRC cells^65,66^. Clinical studies have reported that high expression of Beclin-1 in human advanced colorectal cancer tumors is negativity correlated with the survival rate of patients^67^. These data suggest that inhibition of Beclin-1 by GLP may serve as autophagy-independent mechanism underlying the anti-cancer effects of GLP in CRC cells. However, more studies are needed to definitively determine this hypothesis by using RNAi method that completely deletes Beclin-1 in CRC cells in future.

MAPK has been shown to play an important role in autophagy regulation^28^. However, the precise role of MAPK/ERK in autophagy initiation or maturation has not been defined and yields controversial results^28^. Accumulating evidence indicates that impaired autophagy through disruption of lysosomal function can be triggered by drug-induced activation of MAPK/ERK and thus increases cell death^68,69^. Sustained activation of MAPK/ERK has been shown to inhibit the maturation step of the autophagy process^68^. Activation of MAPK/ERK signaling pathway has also been reported to impair autophagy by promoting degradation of Fork head Box O1 (FOXO1) in cancer cells^70^. FOXO1 has been shown to be a protein involved in the dynamic control of autophagy, which might be essential for maintenance of autophagic flux in cancer cells and neurons^71–74^. It will be interesting to examine whether effect of GLP on MPAK/ERK activation is through targeting FOXO1 in CRC cells in future. Our study suggests that GLP-induced initiation of autophagy and apoptosis is MAPK/ERK-dependent. However, whether MAPK/ERK plays a role in GLP-induced autophagic flux disruption also need to be further examined in future studies.

One discrepancy in our study is that although autophagy flux was inhibited both in vitro and in vivo, GLP failed to increase apoptosis index in CRC tumor samples. One explanation could be that cancer progression in vivo is largely influenced by the tumor microenvironment^75^. Previously, we reported that GLP induced significant increase of necrosis, another form of cell death, in HCT116 xenograft tumors^11^. Both hypoxia and immunity within the tumor play a role that determine how autophagy, apoptosis, and necrosis coordinate with each other^75^. It will be interesting in future to dissect the specific role of GLP-induced autophagy inhibition in cell death in vivo.

In summary, our study demonstrates that GLP triggers apoptosis in CRC by suppressing autophagic flux and inducing autophagosome accumulation. We provide insights into the molecular mechanisms by which GLP elicits the anti-cancer effects against CRC. Our findings provide a basis for potential use of GLP as an adjuvant or direct autophagy inhibitor for CRC treatment. Our results also suggest that increasing the accumulation of autophagosomes by anti-cancer agents may have therapeutic value for CRC.

## Materials and methods

### Materials

#### Cell culture and reagents

Human colon cancer cell lines HT-29 and HCT116 were obtained from American Type Culture Collection (ATCC, VA, USA). Cells were maintained in a humidified atmosphere of 5% CO_2_ at 37 ℃ in Dulbecco’s modified Eagle’s medium (DMEM, Gibco, Grand Island, NY, USA) supplemented with 10% fetal bovine serum (Gemini, Grand Island, NY, USA) and 1% penicillin-streptomycin (Gibco, USA). [3-(4, 5-dimethylthia-zol-2-yl)-2, 5-diphenyltetrazolium bromide] (MTT) and 3-MA were purchased from Sigma-Aldrich (Saint Louis, Missouri, USA). Rap was purchased from Yuanye Biotech (Shanghai, China). CQ was obtained from MedChemExpress Technology (New Jersey, UAS). PD98059 was purchased from Calbiochem (Darmstadt, Germany).

### Preparation of GLP

The powder of sporodum-broken spores of *G. lucidum* was obtained from Shouxiangu Institute of Rare Medicine Plant (Wuyi, Zhejiang, China). GLP from the sporodum-broken spores of *G. lucidum* was extracted by hot water extraction method as described before^11^. Briefly, 5 g power of sporodum-broken spores of *G. lucidum* was placed in 100 ml of ultrapure water, lipid was first removed as described before^76^ and then oscillated (300 rpm) at 70 ℃ for 12 h. However, the proportion of residual lipid and protein contents were not determined at present. The solution was centrifuged at 4000 rpm for 15 min to remove insoluble materials, and further purified to remove protein using Sevage method as described before^76^, and centrifuged again to collect the supernatant. The supernatant was then concentrated and freeze-dried using H051 freeze dryer (LaboGene, Lynge, Denmark) to obtain GLP for subsequent experiments.

### Cell viability assay

HT-29 and HCT116 cells were seeded in 96-well plates at a density of 1 × 10^4^ cells in 200 μl of serum-containing DMEM medium until cells reach 40–50% confluence. To evaluate the cytotoxic effect of GLP on cells, HT-29 and HCT116 cells were treated with different concentrations (0, 2.5, 5, and 10 mg/ml) of GLP for 24, 48, and 72 h. For mechanistic studies, cells were either pre-treated or co-incubated with 3-MA (5 mM), PD98059 (20 μM), Rap (2 μM), or CQ (10 μM) for certain time in the presence or absence of GLP as indicated in figure legends. After incubation, MTT solution (5 mg/ml) was added to the DMEM and incubated for an additional 4 h. Then, supernatants were removed and the purple formazan crystals were dissolved in 150 μl DMSO and absorbance was measured at 490 nm using a microplate reader (Bio-Tek Instrument Inc., Winooski, VT, USA). The percentage of viability was expressed as (ODsample−ODblank)/(ODcontrol−ODblank) × 100%, where OD is the absorbance.

### Quantification of GFP-LC3 puncta

HT-29 and HCT116 cells were seeded in CELLview^TM^ glass bottom dish (USA Scientific, Ocala, FL, USA) at a density of 1 × 10^3^ cells in 1 ml of serum-containing DMEM until cells reach 30% confluence. The cells were transfected with adenovirus GFP-LC3 (Hanbio Biotech, Shanghai, China), and then treated with GLP or indicated chemicals for additional 24 h. Cells were observed under a laser scanning confocal microscope (LSM880, Carl Zeiss, Germany). The average number of GFP-LC3 puncta per cell was counted with at least 100 cells for each cell line.

### Transmission electron microscope

HT-29 cells were seeded in 6-well plates at a density of 2 × 10^5^ cells in 2 ml of serum-containing DMEM until cells reach 40–50% confluence, Cells were then treated with GLP (5 mg/ml), 3-MA (5 mM), CQ (10 μM), GLP/3-MA, or GLP/CQ for 24 h. The cells were washed with PBS and harvested by trypsinization, and then the cells were fixed with 2.5% glutaraldehyde for overnight at 4 ℃. Next day, glutaraldehyde was removed and washed three times in the PBS for 15 min at each step. Then, cells were first embedded in agarose gel and fixed with 1% OsO_4_ in PBS for 1 h, OsO_4_ was removed and washed three times in the PBS for 15 min at each step. The cells were then dehydrated through an ascending series of ethanol (30, 50, 70, 80, 90, 95%) for 15 min at each step, then dehydrated by 100% ethanol for 20 min. Next, the cells were transferred to absolute acetone for 20 min, and the cells were placed in 1:1 mixture of absolute acetone and Spurr resin for 1 h at room temperature, then transferred to 1:3 mixture of absolute acetone and the Spurr resin for 3 h and further transferred to Spurr resin mixture for overnight. Samples were placed in Eppendorf tube containing Spurr resin and heated at 70 ℃ for more than 9 h. The samples were sectioned with Leica EM UC7 ultramicrotome (Leica, Nussolch, Germany). After staining with uranyl acetate and alkaline lead citrate for 10 min, respectively, samples were observed with a Hitachi H-7650 transmission electron microscope (Hitachi, Tokyo, Japan). All images were acquired by a Gatan SC1000 (Model 832) CCD camera (Gatan Inc, Pleasanton, CA, USA).

### mRFP-GFP-LC3 adenovirus translocation and colocalization analysis

HT-29 and HCT116 cells were seeded in CELLview^TM^ glass bottom dish (USA Scientific) at a density of 1 × 10^3^ cells in 1 ml of serum-containing DMEM until cells reach 30% confluence. Cells were then transfected with the mRFP-GFP-LC3 adenovirus (multiplicity of infection = 300) for 6 h in DMEM supplemented with 10% FBS and 1% p/s. DMEM were removed and washed twice with PBS. Cell were incubated for 24 h in serum-containing DMEM. Cells were treated with GLP (5 mg/ml), Rap (2 μM), or CQ (10 μM) for 24 h. After incubation, image acquisition was done using a laser scanning confocal microscope (LSM880, Carl Zeiss, Germany). mRFP and GFP expressed in mRFP-GFP-LC3 tandem fluorescent protein adenovirus were used to track LC3. As GFP fluorescent protein is sensitive to acidity, the GFP fluorescence was quenched after the fusion of autophagosome and lysosome. Therefore, the weaken of GFP could indicate the fusion of autophagosome and lysosome to form autolysosome. When red and green fluorescence merged, the yellow spots represent autophagosomes and red spots represent autolysosomes. When autophagy occurs, there will be more red spots than yellow spots, and when the fusion of autophagosomes-lysosomes is damaged, the yellow spots are dominant^22,23^.

### Measurement of intralysosomal acidity

The LysoSensor^TM^ Green DND-189 reagent (Yeasen Biotech, Shanghai, China) exhibits an acid-dependent increase in fluorescence intensity. Cell staining was performed according to the manufacturer’s protocol. Briefly, cells were treated with GLP (5 and 10 mg/ml) or CQ (10 μM) for 24 h. The cells were then washed twice with PBS and added a final concentration of 2 uM LysoSensor reagent, and further incubated for 20 min at room temperature (RT) in the dark. The fluorescence intensity was observed under an Ti-S inverted fluorescence microscope (Nikon, Tokyo, Japan).

### RNA isolation and quantitative real-time PCR

Total RNA isolation was performed using EASYspin reagent Kit (Aidlab Biotech, Beijing, China), following the manufacturer’s protocol. Both the quality and quantity of total RNA were analyzed by the NanoDrop 2000c spectrophotometer (Thermo Scientific, Grand Island, NY, USA). First-strand cDNA was synthesized from 1 µg of total RNA using iScript Reverse transcription supermix (Bio-Rad, Hercules, CA, USA). qRT-PCR analysis was performed using SYBR Green master mix (Bio-Rad) on CFX96 Real-Time PCR system (Bio-Rad). Primers were designed with open-sourced software Primer3Plus (Cambridge, MA, USA). Beta-Actin was used as the reference gene. The relative expression of mRNA was calculated as follows: ΔCt = Ct (sample)−Ct (β-Actin), ΔΔCt (sample) = ΔCt (sample)−ΔCt (calibrator). The fold change of mRNA was calculated according to the relative quantification (2^−ΔΔCt^). Primer sequences of qRT-PCR reactions are shown in Supplementary Table 1.

### Western blotting

Polyclonal LC3, Beclin-1, p62, MAPK/ERK, p-MAPK/ERK, mTOR, p-mTOR (Ser2448), AMPKα, p-AMPKα (Ser485), PARP, β-Actin, and monoclonal CTSB antibodies were purchased from Cell Signaling Technology (Danvers, MA, USA). Monoclonal CTSD and LAMP-2 antibodies were purchased from Abcam (Cambridge, England). Cells were lysed in ice-old RIPA buffer (PBS, 1% Igepal, 10% SDS, 0.5% Sodium Deoxycholate) supplemented with complete protease inhibitors and total protein concentrations were measured by bicinchoninic acid (BCA) protein assay kit (Pierce, Rockford, IL, UAS). An equal amount of total protein (40 μg) was resolved by SDS–PAGE and transferred to PVDF (polyvinylidene difluoride) membrane (MilliporeSigma, USA). After blocking with 5% nonfat milk in 1× TBST (Tris-buffered saline, 50 mM of Tris, 150 mM of NaCl, pH 7.5, 0.1% Tween-20) at RT for 1 h, the membranes were probed with the following antibodies: LC3, Beclin-1, p62, p-mTOR (Ser2448), t-mTOR, p-ERK, t-ERK, p-AMPKα (Ser485), t-AMPKα, CTSB, CTSD, PARP, LAMP-2, and β-Actin at indicated concentrations as listed in Supplementary Table 2. Membranes were incubated with primary antibody for overnight at 4 ℃. After washing with TBST, the membranes were incubated with secondary anti-rabbit antibody (1:2000) at RT for 1 h. Then signals were captured using western lightning Plus ECL enhanced chemiluminescence substrate from PerkinElmer (Waltham, MA, USA), and detected using Minichemi TM610 chemical imaging System (Beijing, China). The quantitative of optical density was analyzed using ImageJ 1.41 software (Bethesda, MD, USA).

### Flow cytometric analysis of apoptosis

Flow cytometric analysis was used for apoptosis assay. Cells (2 × 10^5^) were seeded in 6-well plate per well and treated with GLP (5 mg/ml), 3-MA (5 mM), CQ (10 μM), GLP/3-MA or GLP/CQ for 24 h. When analyzing the effect of PD98059 and Rap on GLP-induced apoptosis, cells were starved overnight, then pre-treated with PD98059 (20 μM) and Rap (2 μM) for 2 h, respectively, and then treated for 24 h in the absence or presence of GLP (5 mg/ml). The cells were washed with PBS, harvested by trypsinization and washed twice with PBS. The cells were then stained with propidium iodide (PI) and annexin V-FITC using FITC Annexin V Apoptosis Detection Kit (BD Pharmingen, San Diego, CA, USA) according to manufacturer’s instruction. Briefly, cells were re-suspended with 1× binding buffer at a density of 1 × 10^6^ cells/ml. Then added PI and Annexin V-FITC (5 μl) to cell suspension (100 μl) before further incubation for 15 min at RT in the dark. Stained cells were diluted with 1× binding buffer and analyzed by Guava EasyCyte flow cytometer (Merck KGaA, Darmstadt, Germany). The percentage of Annexin V^+^/PI^−^ (early apoptosis), Annexin V^+^/PI^+^ (late apoptosis), and Annexin V^−^/PI^+^ (necrosis) cells was analyzed on the basis of manufacture’s instruction (BD Pharmingen). Data was represented as rate of total apoptotic cells with both early and late apoptotic rate indicated.

### Xenograft mouse model

All the experimental procedures were performed according to the Guide for the Care and Use of Laboratory Animals of the National Institutes of Health. All procedures of the experiment are in line with the Animal Welfare Act Regulations. This study was approved by the Committee on the Ethics of Animal Experiments of Zhejiang Chinese medical University (Permit Number: SYXK 2012-0002). Male BALB/C nude mice (four-week-old) were kept in specific pathogen free (SPF) environment at the experimental animal center at the Zhejiang Chinese Medical university. After 2 week of adaptation, the mice were randomly divided by weight into treatment and control groups (*n* = 10 per group): control group (saline), low dose group (150 mg/kg), and high dose group (300 mg/kg). HT-29 cells (5 × 10^6^ cells in 200 ul PBS) were implanted into the right flank of nude mouse.

The day after implantation of cancer cells, the mice were treated with GLP by gavage per day in low and high dose groups, while the control group mice were treated with saline per day. Body weights and tumor volume were measured twice a week and distinct tumors were measured in length and width using a digital vernier caliper (0.01 mm). Tumor volume was assessed according to the formula V (mm^3^) = length × width^2^/2. Six weeks after injection, all mice were sacrificed. At the end of study, 1–3 mice of each group did not grow tumor: control (*n* = 1); 150 mg/kg of GLP (*n* = 2); 300 mg/kg of GLP (*n* = 3). In addition, there were 2 mice in control group and 1 mouse in low dose group developed large tumor (>1.8 cm) very early during the study, and thus, these mice were sacrificed early and removed from study. Therefore, data from 7 mice of each group were analyzed. After necropsy, xenograft tumors were carefully excised and weighed. The tumor tissues were snap frozen in liquid nitrogen and stored at −80 ℃ for subsequent analysis.

### Statistical analysis

All results presented are representatives from at least three independent experiments. Data are expressed as mean ± standard error (SE) of indicated number of experiments. For comparisons between two groups, a Student *t-*test was used. And for analysis with multiple comparisons, one-way analysis of variance (ANOVA) with Dunnett’s correction or Bonferroni’s correction for pairwise comparison was used. A value of *P < 0.05 was considered as statistically significant. All analyses were performed using Graphpad Prism 5.0 (GraphPad Software, Inc., La Jolla, CA, USA).

## Supplementary information

Supplementary Materials
