# Supplementary material for: Autophagic flux disruption contributes to Ganoderma lucidum polysaccharide-induced apoptosis in human colorectal cancer cells via MAPK/ERK activation
Source: Cell Death Dis. 2019 Jun 11;10(6):456. doi: 10.1038/s41419-019-1653-7 (PMC6560101; doi:10.1038/s41419-019-1653-7)
Supplement: Supplementary file 1 — Supplementary Materials [file 41419_2019_1653_MOESM1_ESM.pdf]

**Supplementary Information**

**Supplementary method**

**TUNEL staining and apoptotic index measurement**

Apoptotic index was determined by TUNEL staining. Tumor samples were harvested and fixed in 10% formalin for 24 h. The fixed tumor tissues were embedded in paraffin, and 5-μm sections were cut for TUNEL staining using TUNEL assay kit (Roche Applied Sciences, Basel, Switzerland) according to manufacturer's instruction. Approximately 300 cells per field of each section and 3 fields of each section were counted. The number of TUNEL-positive cells detected in tumor samples were calculated to determine the apoptotic index.

**Supplementary Figure 1**

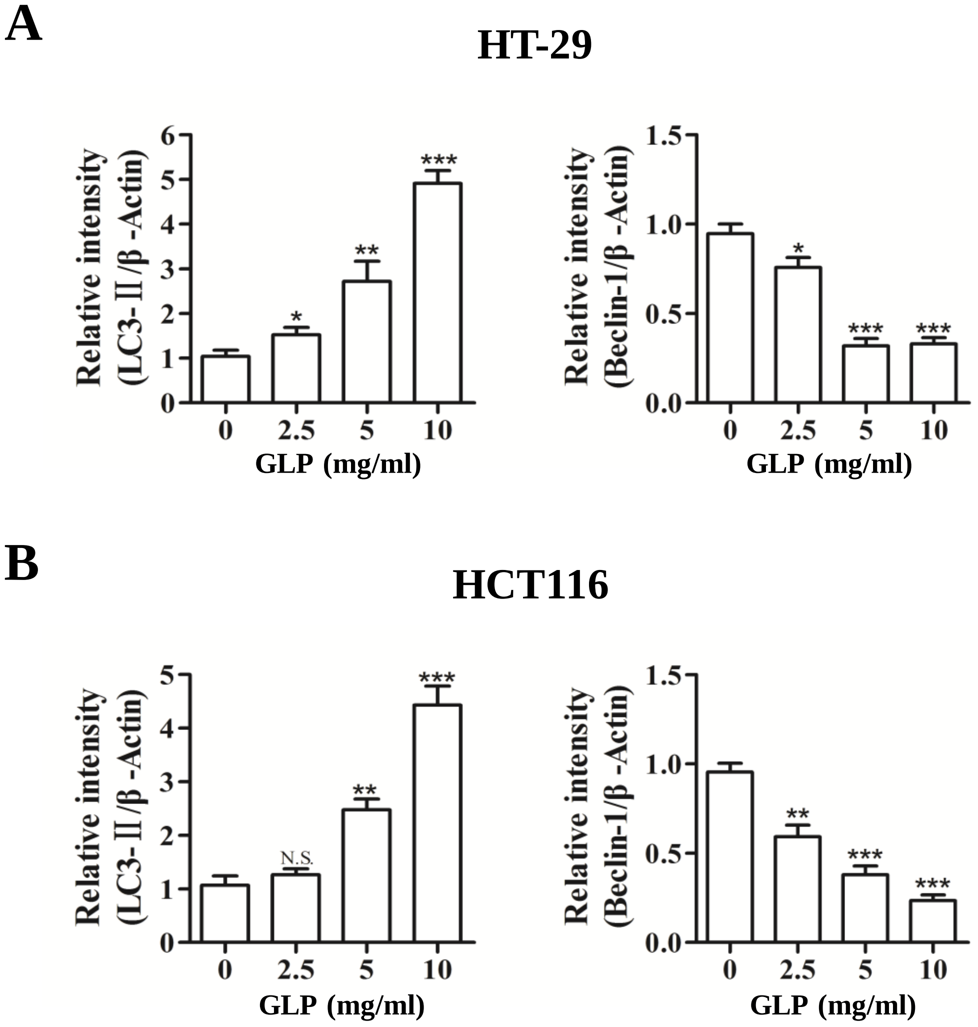

## Supplementary Figure 2

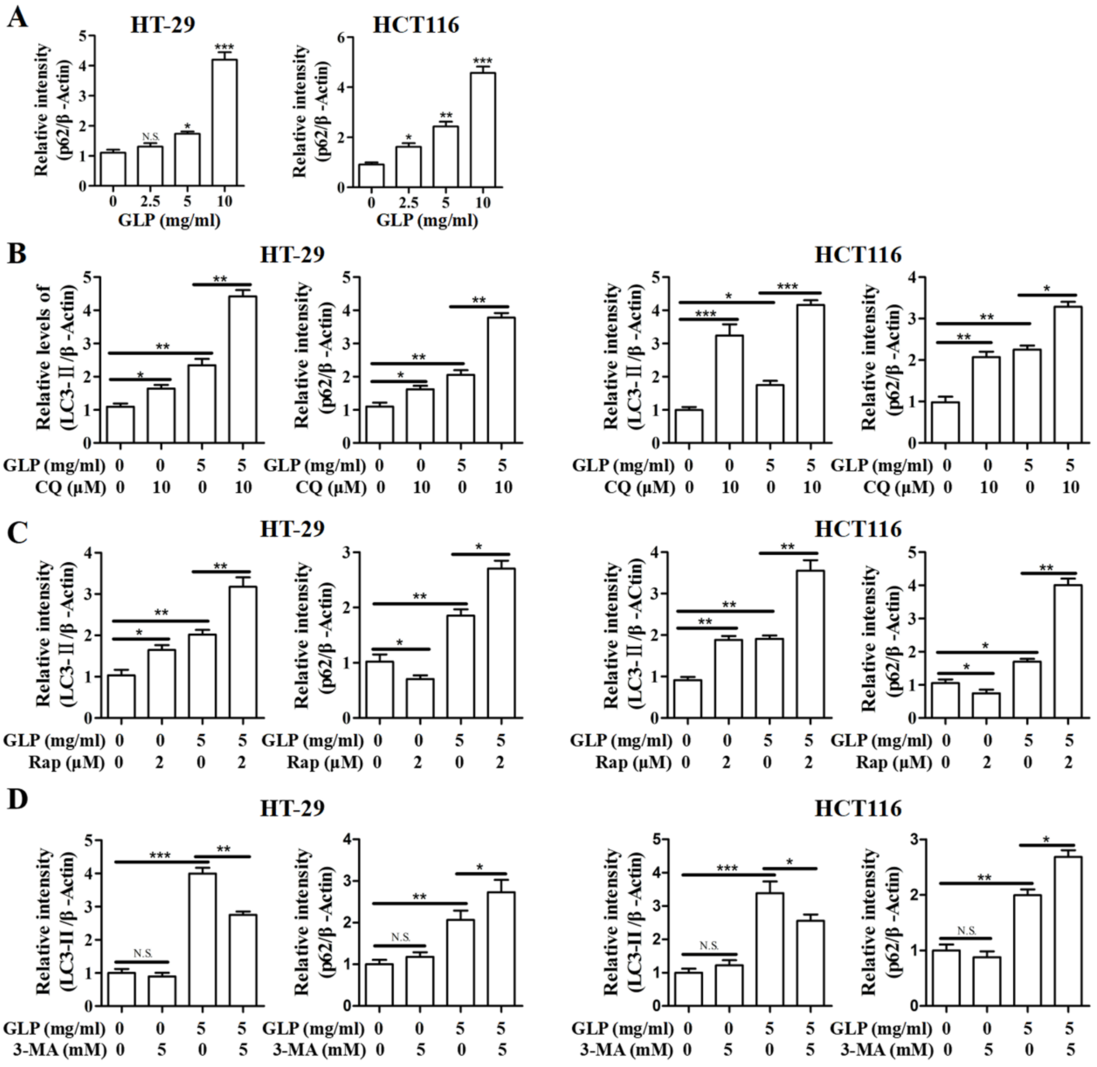

**Supplementary Figure 3**

A

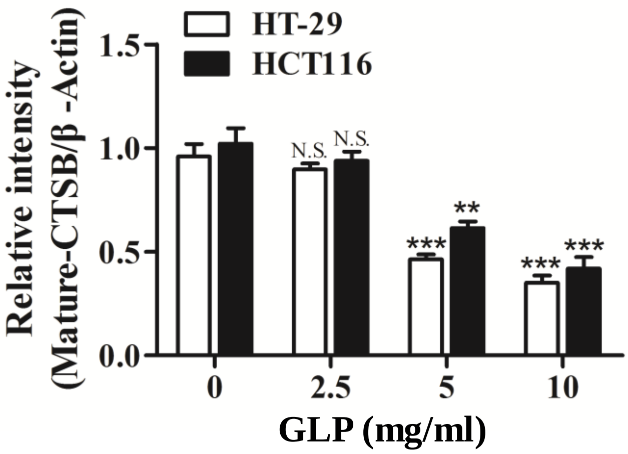


B

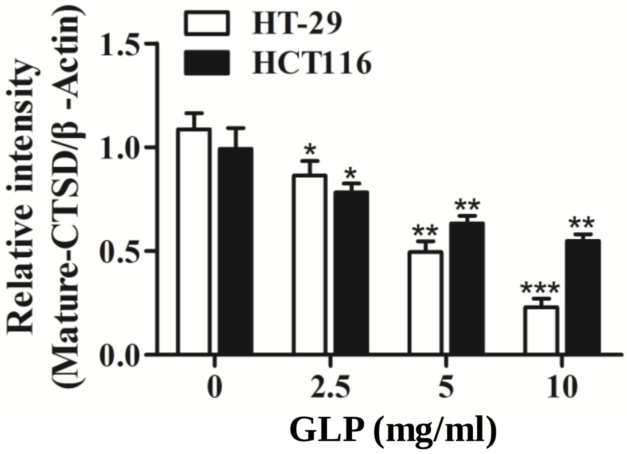

# Supplementary Figure 4

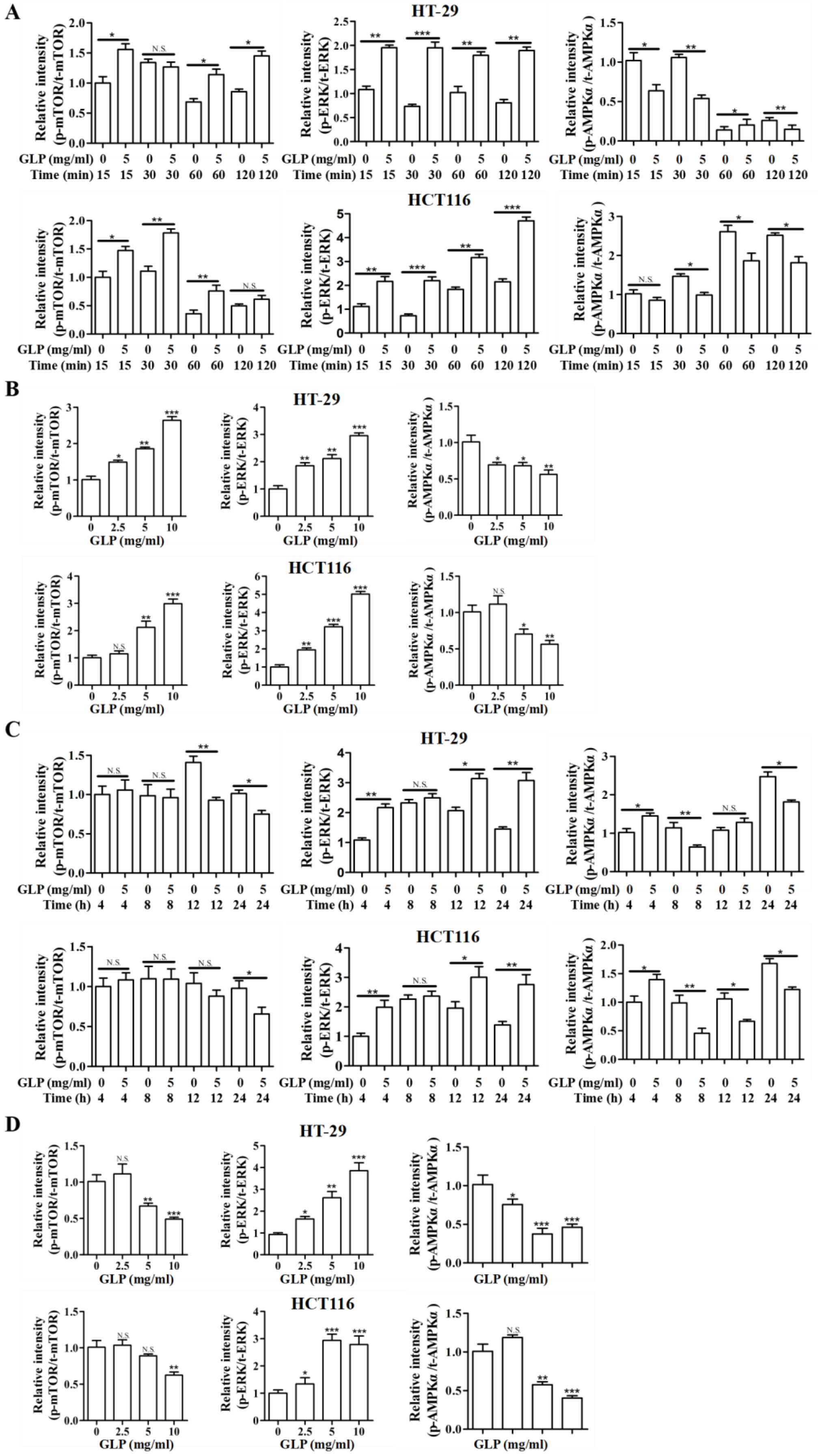

**Supplementary Figure 5**

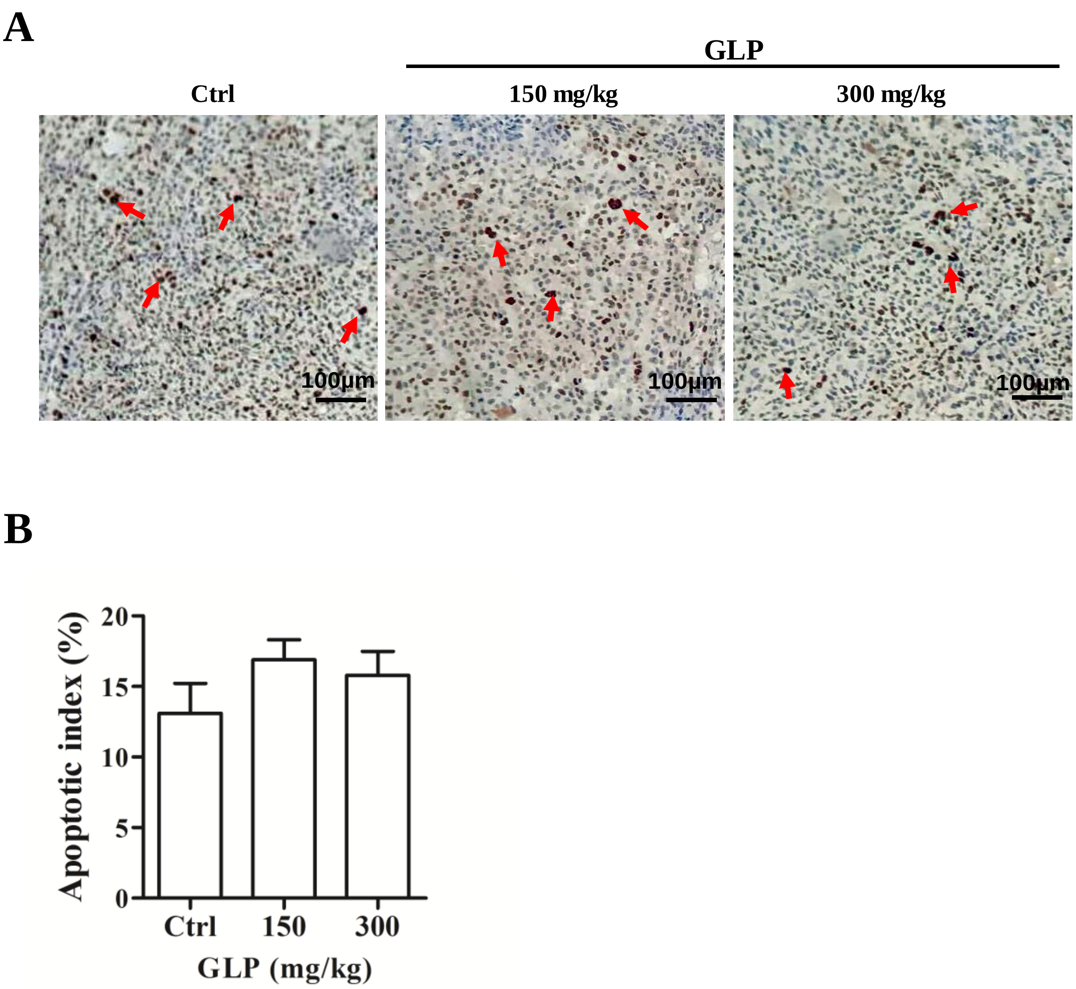

## Supplementary Figure 6

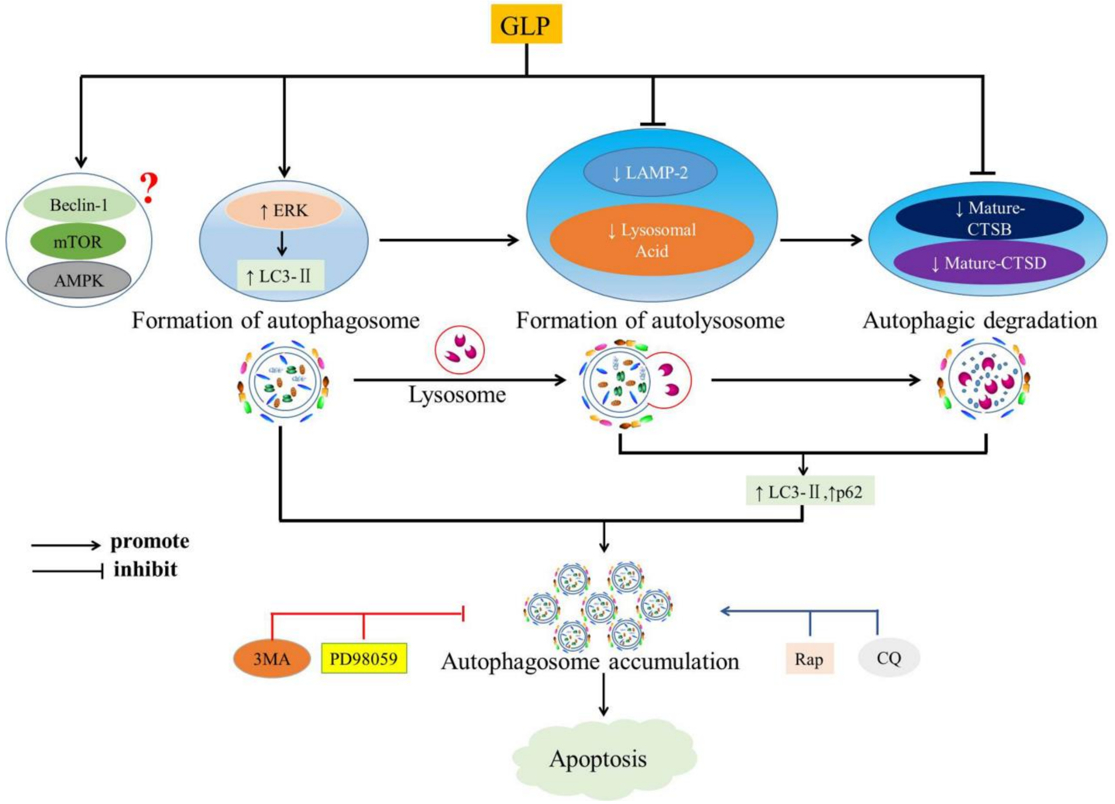

**Spplementary Table 1 Primer sequence of qRT-PCR reactions**

| Primers | | Sequences |
|---|---|---|
| LC3-II | Forward | GATGTCCGACTTATTCGAGAGC |
| | Reverse | TTGAGCTGTAAGCGCCTTCTA |
| Beclin-1 | Forward | AGCTGCCGTTATACTGTTCTG |
| | Reverse | ACTGCCTCCTGTGTCTTCAATCTT |
| p62 | Forward | GAACTCCAGTCCCTACAGATGCC |
| | Reverse | CGGGAGATGTGGGTACAAGG |
| CTSB | Forward | AACACGTCACCGGAGAGATGA |
| | Reverse | CCCAGTCAGTGTTCCAGGAGTT |
| CTSD | Forward | GGCTCTGTGGAGGACCTGATTG |
| | Reverse | CGATGCCAATCTCCCCGTGTA |
| LAMP-2 | Forward | ACAACAGTGGATCAGACAGTACG |
| | Reverse | AGCAGCAAGCATCAGTTCTTC |
| β-Actin | Forward | CTGGAACGGTGAAGGTGACA |
| | Reverse | AAGGAACTTCCTTGAACAATGCA |

**Supplementary Table 2 Sources and dilutions of primary antibodies for WB**

| Antibody | Supplier and catalog no. | Dilution |
|---|---|---|
| LC3-I/II | Cell signaling, 4108 | 1:1000 |
| p62 | Cell signaling, 8025 | 1:1000 |
| Beclin-1 | Cell signaling, 3495 | 1:1000 |
| p-ERK | Cell signaling, 9101s | 1:1000 |
| ERK | Cell signaling, 9102s | 1:1000 |
| p-mTOR | Cell signaling, 5536p | 1:1000 |
| mTOR | Cell signaling, 2983p | 1:1000 |
| p-AMPKα | Cell signaling, 2537 | 1:1000 |
| AMPKα | Cell signaling, 4184 | 1:1000 |
| CTSB | Cell signaling, 31718 | 1:1000 |
| CTSD | Abcam, ab75852 | 1:5000 |
| LAMP-2 | Abcam, ab199946 | 1:2000 |
| PARP | Cell signaling, 9542 | 1:1000 |
| β-Actin | Cell signaling, 4967 | 1:1000 |

*WB* Western Blotting

**Supplementary figure legends**

**Supplementary Fig. 1 Densitometric analysis of western blots for Fig. 1e. a** Quantitative analysis of LC3-II and Beclin-1 expression in HT-29 cells. **b** Quantitative analysis of LC3-II and Beclin-1 expression in HCT116 cells. The relative intensities of LC3-II and Beclin-1 were calculated after normalization against β-Actin. Data are presented as the mean ± SE from three independent experiments. *P < 0.05; **P < 0.01; ***P < 0.001 compared with untreated cells. N.S., No significance.

**Supplementary Fig. 2 Densitometric analysis of western blots for Fig. 2a, 2c, 2d, and 2e. a** Quantitative analysis of p62 expression in HT-29 and HCT116 cells. **b** Quantitative analysis of LC3-II and p62 expression in HT-29 and HCT116 cells upon CQ treatment. **c** Quantitative analysis of LC3-II and p62 expression in HT-29 and HCT116 cells upon Rap treatment. **d** Quantitative analysis of LC3-II and p62 expression in HT-29 and HCT116 cells upon 3-MA treatment. The relative intensities of LC3-II and p62 were calculated after normalization against β-Actin signal intensity. Data are presented as the mean ± SE from three independent experiments. *P < 0.05; **P < 0.01; ***P < 0.001 compared with indicated samples. N.S., No significance.

**Supplementary Fig. 3 Densitometric analysis of western blots for Fig. 4c. a** Quantitative analysis of Mature-CSTB expression in HT-29 and HCT116 cells. **b** Quantitative analysis of Mature-CSTD expression in HT-29 and HCT116 cells. The relative intensity of mature-CSTB and mature-CSTD were calculated after normalization against β-Actin. Data are presented as the mean ± SE from three independent experiments. *P < 0.05; **P < 0.01; ***P < 0.001 compared with untreated

cells. N.S, No significance.

**Supplementary Fig. 4 Densitometric analysis of western blots for Fig. 5a, 5b, 5c and 5d. a** Quantitative analysis of p-mTOR, p-MAPK/ERK and p-AMPKα expression in HT-29 and HCT116 cells upon treatment with GLP (5 mg/ml) for 15, 30, 60, and 120 min. **b** Quantitative analysis of p-mTOR, p-MAPK/ERK and p-AMPKα expression in HT-29 and HCT116 cells upon treatment with indicated concentrations of GLP for 1 h. **c** Quantitative analysis of p-mTOR, p-MAPK/ERK and p-AMPKα expression in HT-29 and HCT116 cells upon treatment with GLP (5 mg/ml) for 4, 8, 12, and 24 h. **d** Quantitative analysis of p-mTOR, p-MAPK/ERK and p-AMPKα expression in HT-29 and HCT116 cells upon treatment with indicated concentrations of GLP for 24 h. The relative intensities of p-mTOR, p-MAPK/ERK and p-AMPKα were calculated after normalization against p-mTOR, p-MAPK/ERK and p-AMPKα, respectively. Data are presented as the Mean ± SE from three independent experiments. *P < 0.05; **P < 0.01; ***P < 0.001 compared with untreated cells. N.S., No significance.

**Supplementary Fig. 5 TUNEL detection of apoptosis index in xenograft tumor samples after treating with GLP. a** TUNEL staining of xenograft tumor sections. The arrows indicate representative, but not all, apoptotic cells. Images are representative of 3 samples per treatment group. Scale bar: 100μm. **b** Apoptosis index as calculated by % TUNEL positive cells in tumor samples. Data are represented as Mean ± SE from 3 fields from each sample per group (n=9).

**Supplementary Fig. 6** Working model of molecular mechanisms by which GLP exerts its anticancer activity and regulate autophagy in CRC HT-29 and HCT116 cells.
